# Developmental stage-dependent changes in kaempferol derivatives and associated antioxidant, antibacterial, and quorum sensing inhibitory activities in *Hylotelephium verticillatum* leaves

**DOI:** 10.1016/j.fochx.2026.104520

**Published:** 2026-09-25

**Authors:** Neil Patrick Uy, Reyna Marie Therese Sanchez, Sang-Yun Lee, Jonie Yee, Chung-Ho Choi, Sanghyun Lee

**Affiliations:** a Department of Plant Science and Technology, Chung-Ang University, Anseong 17546, Republic of Korea; b Department of Mathematics and Natural Sciences–Biology, Cebu Institute of Technology–University, Cebu 6000, Philippines; c Department of Biology, University of San Carlos, Cebu 6000, Philippines; d Gyeonggi-do Forestry Environment Research Center, Osan 18118, Republic of Korea; e Natural Product Institute of Science and Technology, Anseong 17546, Republic of Korea

**Keywords:** *Hylotelephium verticillatum*, Leaf ontogeny, Succulent crops, Kaempferol glycosides, Antibacterial activity

## Abstract

This study investigated the phytochemical profiles and bioactivities of ethanolic extracts from the first and second true leaves (FLH and SLH) of *Hylotelephium verticillatum* during early development. Total phenolic content (TPC), total flavonoid content (TFC), antioxidant, antibacterial, and quorum sensing (QS) inhibitory activities were evaluated, and major flavonoids were characterized using UPLC-Q-orbitrap-ESI-MS/MS and HPLC. SLH exhibited significantly higher TPC than FLH, whereas TFC showed no significant difference. Despite lower TPC, FLH demonstrated stronger ABTS radical scavenging, antibacterial, and QS inhibitory activities (based on diffusion assays). Phytochemical analysis revealed that kaempferol derivatives were the predominant constituents and displayed clear developmental stage-dependent variations. Correlation and hierarchical clustering analyses indicated that biological activities were more closely associated with specific flavonoid constituents than with overall phenolic accumulation. These findings demonstrate that early leaf development significantly influences flavonoid composition and bioactivities in *H. verticillatum*, highlighting the functional importance of kaempferol derivatives in this edible species.

## 1 Introduction

Plants produce a wide variety of secondary metabolites, including phenolic compounds and flavonoids, which play crucial roles in growth, defense, and adaptation to environmental stresses (Bajaj et al., 2025). These metabolites are well known for their diverse biological activities, particularly their antioxidant and antimicrobial properties, and have attracted considerable interest as natural alternatives to synthetic agents in pharmaceutical and food applications. In the food industry, plant-derived phenolics are highly valued not only for their health-promoting functional properties but also as natural preservatives capable of inhibiting foodborne pathogens and preventing lipid oxidation. Beyond their direct antimicrobial effects, many phenolic compounds and flavonoids have been shown to interfere with bacterial communication systems such as quorum sensing (QS), thereby suppressing virulence and biofilm formation without imposing strong selective pressure for the development of antimicrobial resistance (Lima et al., 2023).

The accumulation of these metabolites is highly dynamic and is influenced by factors such as developmental stage, tissue type, and environmental conditions (Liu et al., 2025; Park et al., 2024). Leaves, in particular, undergo extensive metabolic changes during early development, which can substantially alter both the composition and abundance of secondary metabolites. From a dietary and agricultural perspective, these early developmental phases often coincide with the optimal harvesting window for leafy vegetables, where young tissues possess desirable sensory attributes such as tenderness and palatability. The first and second true leaves represent critical stages in plant establishment and survival, during which chemical defense systems may be especially important (Wiggins et al., 2016). Despite the ecological and physiological significance of these early developmental phases, the relationship between leaf ontogeny, phytochemical accumulation, and bioactivity remains poorly understood.

The genus *Hylotelephium* (Crassulaceae), formerly classified within the genus *Sedum*, comprises succulent perennial species that are widely distributed throughout temperate regions (Cheshmedzhiev & Marinov, 2019). Notably, several species within this genus have a history of traditional dietary use, being consumed fresh as wild edible greens or salad vegetables, particularly during their tender young growth stages (Byeon et al., 2014; Chung et al., 2016). Species within this genus are known to contain a diverse array of phytochemicals, including flavonoids and phenolic acids, which are believed to contribute to their reported antioxidant and medicinal properties (Li et al., 2024). Consequently, characterizing their chemical profiles during the edible phase is critical to validating their nutritional worth and potential as functional foods. Nevertheless, existing research on *Hylotelephium* has focused predominantly on taxonomy, horticultural characteristics, and genomic studies, whereas its phytochemical composition and biological activities have received comparatively little attention (Funamoto et al., 2007, Kim and Kim, 2020). Among the species in this genus, *H. verticillatum* remains particularly understudied, with limited information available regarding its metabolite profile and bioactive potential. Moreover, no previous studies have investigated how early leaf development influences the accumulation of bioactive metabolites and the resulting biological activities in this species.

In this study, we investigated the influence of early leaf developmental stages on the phytochemical composition and biological activities of *H. verticillatum* to evaluate its nutritional value and functional food potential. Specifically, the first and second true leaves (FLH and SLH, respectively) were compared to determine how developmental progression affects the accumulation of phenolic and flavonoid compounds and their associated radical scavenging, antibacterial, and QS inhibitory activities. To this end, comprehensive metabolite profiling was performed using UPLC-Q-orbitrap-ESI-MS/MS, and major flavonoids were quantified by HPLC analysis. These approaches enabled the identification and quantification of key metabolites potentially associated with the observed biological activities.

To the best of our knowledge, this study is the first to comprehensively examine developmental stage-dependent changes in phytochemical composition alongside radical scavenging, antibacterial, and QS inhibitory activities in *H. verticillatum*. By integrating metabolite profiling with bioactivity assessments, this work provides new insights into the relationship between early leaf development, flavonoid accumulation, and the functional properties of this species.

## 2 Materials and methods

### 2.1 Plant materials and cultivation conditions

In mid-September 2024, the seeds of *H. verticillatum* (L.) H. Ohba were collected from an experimental field in Osan managed by the Gyeonggi-do Forestry Environment Research Center (GFERC). Species authentication was conducted by C.-H. Choi at the GFERC, where voucher specimens were subsequently cataloged and stored under accession number GFERC 202505. Cultivation commenced in early April 2025 at the identical field plot, yielding a germination success rate of roughly 70%. Foliar collection was executed 20 days post-germination once true leaf morphology stabilized; at this juncture, the first true leaves (FLH) and second true leaves (SLH) were systematically isolated for downstream processing (Fig. 1). Leaves were dried at 37 °C for 2 weeks before being finely powdered using a mechanical grinder.

**Fig. 1:**
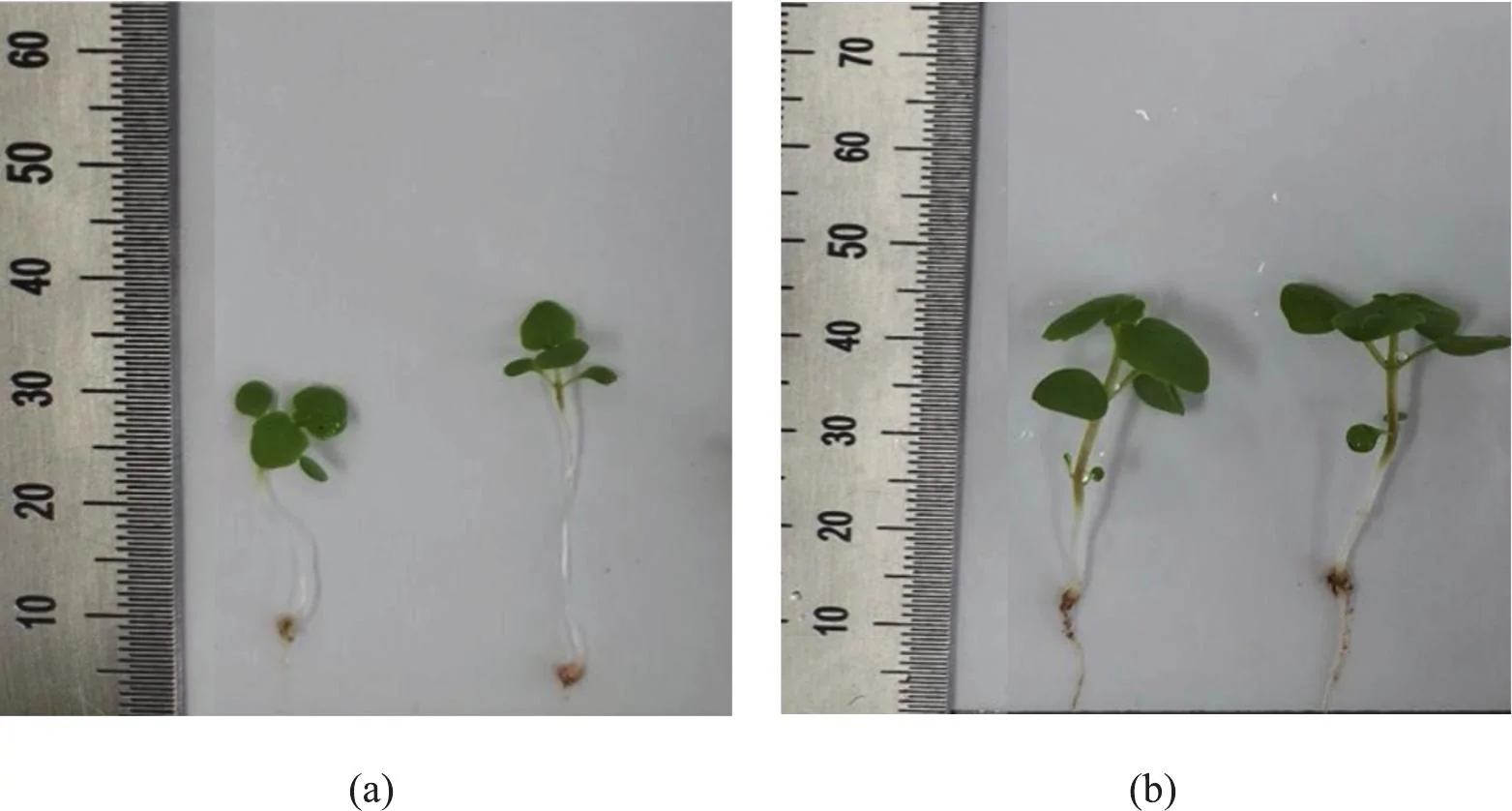
Morphological appearance of FLH (a) and SLH (b) seedlings of *H. verticillatum.*

### 2.2 Chemicals and reagents

All chromatographic analyses were performed using HPLC-grade solvents and reagents. Scharlau (Sentmenat, Barcelona, Spain) supplied the acetonitrile (ACN), and Thermo Fisher Scientific (Waltham, MA, USA) provided the formic acid (FA). Water and methanol (MeOH) sources were manufactured by Honeywell Burdick & Jackson (Charlotte, NC, USA). For extraction protocols, 95% ethanol (EtOH) was procured from Samchun Pure Chemical Co., Ltd. (Pyeongtaek, Gyeonggi, Republic of Korea). Authentic standards, including kaempferol 3,7-di-*O*-rhamnoside (**1**), kaempferol 7-*O*-neohesperidoside (**2**), kaempferol 3-*O*-glucoside (**3**), kaempferol 3-*O*-rhamnoside (**4**), kaempferol 7-*O*-rhamnoside (**5**), and kaempferol (**6**), were provided by the Natural Product Institute of Science and Technology (Anseong, Republic of Korea; www.nist.re.kr) (Fig. 2).

**Fig. 2:**
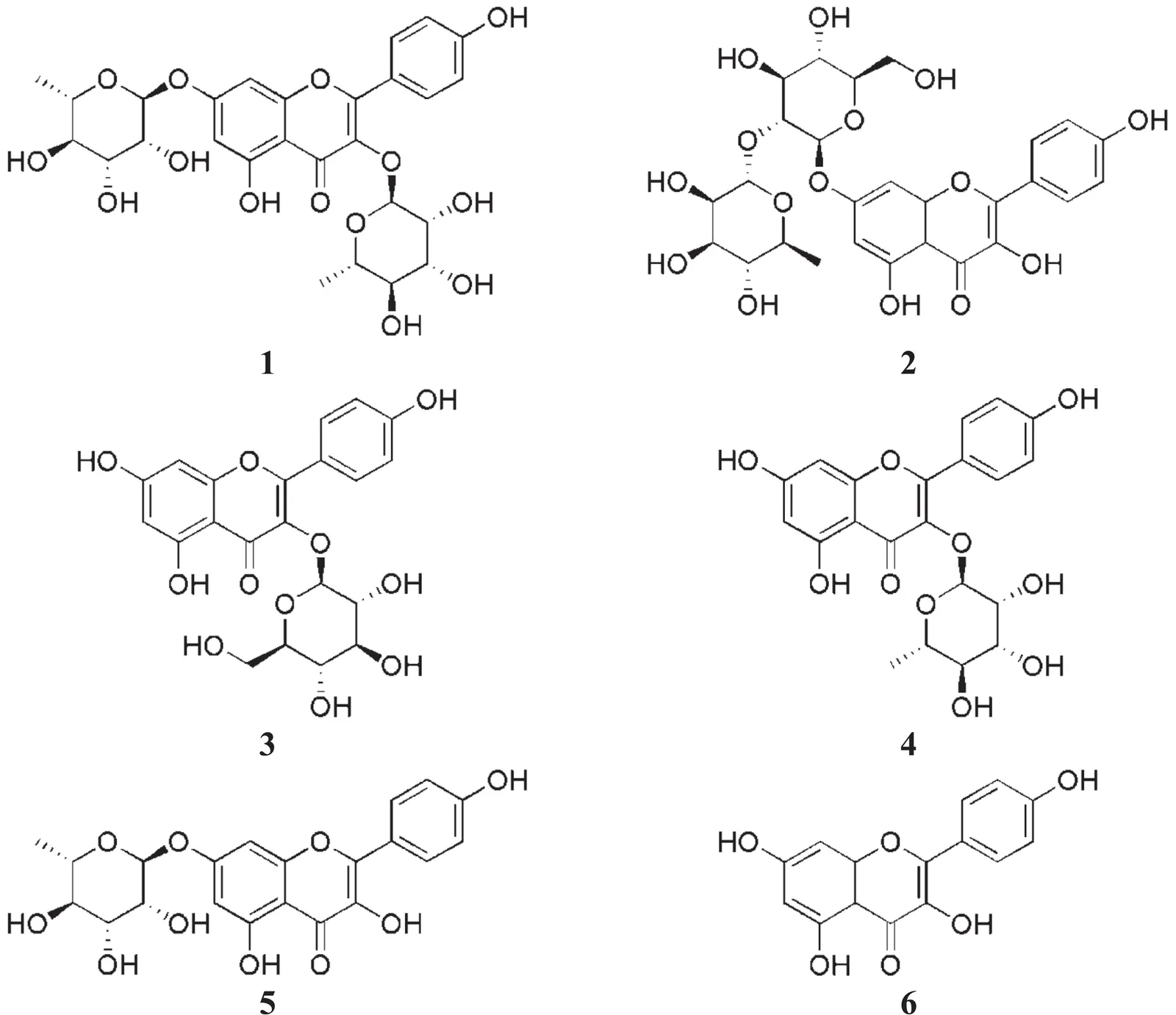
Chemical structures of compounds **1**–**6**: kaempferol 3,7-di-*O*-rhamnoside (**1**), kaempferol 7-*O*-neohesperidoside (**2**), kaempferol 3-*O*-glucoside (**3**), kaempferol 3-*O*-rhamnoside (**4**), kaempferol 7-*O*-rhamnoside (**5**), and kaempferol (**6**).

### 2.3 Crude extraction

Metabolites were extracted from *H. verticillatum* leaves using a reflux extraction method. Briefly, 1 g of finely powdered FLH or SLH sample was placed in a separate extraction flask and mixed with 300 mL of absolute EtOH. Thermal extraction was maintained at 80 °C for a duration of 3 h. To maximize metabolite yields, this exhaustive process was conducted three consecutive times for every sample batch. Filtration steps removed the spent biomass, and the resulting liquid phases were pooled and evaporated to total dryness under reduced pressure to yield the crude solid extracts. Although prolonged heating may affect highly heat-labile compounds, these conditions were selected to provide efficient and reproducible metabolite recovery under controlled reflux conditions.

### 2.4 Determination of total phenolic and flavonoid contents

Total phenolic content (TPC) and total flavonoid content (TFC) of FLH and SLH extracts were determined using modified colorimetric methods (Lee et al., 2026) in 96-well microplates. For TPC analysis, 60 μL of extract was mixed with 40 μL of Folin–Ciocalteu reagent (Sigma-Aldrich, St. Louis, MO, USA), followed by the addition of 100 μL of 7.5% (*w*/*v*) sodium carbonate (Na_2_CO_3_). After incubation in the dark at room temperature for 30 min, absorbance was measured at 760 nm using a microplate reader (Epoch; BioTek, Winooski, VT, USA). Results were expressed as mg tannic acid equivalents per gram of extract (mg TAE/g extract). For TFC analysis, 100 μL of extract was mixed with 100 μL of 2% aluminum chloride (AlCl_3_) solution and incubated at room temperature for 10 min. Absorbance was measured at 430 nm, and TFC values were calculated using a quercetin calibration curve. Results were expressed as mg quercetin equivalents per gram of extract (mg QE/g extract).

### 2.5 Radical scavenging activity

Free radical interception performance was measured through ABTS and DPPH methods with slight alterations (Lee et al., 2026). Assays were performed in triplicate in 96-well configurations, utilizing ascorbic acid as the standard baseline comparison. For the ABTS protocol, 10 μL of sample was combined with 200 μL of an active ABTS+ radical suspension, stored in the dark for 30 min at room temperature, and read at 734 nm. For the DPPH system, 10 μL of extract was added to 200 μL of a 0.2 mM DPPH solution prepared in 95% ethanol. Incubation followed identical dark environment conditions before checking the absorbance values at 514 nm. Scavenging percentages across both metrics were assessed against a negative control to map the dose-response profiles.

### 2.6 Preparation of microbial inocula

For antibacterial assays, *Staphylococcus aureus* and *Escherichia coli* were used as test organisms, whereas *Chromobacterium violaceum* was employed as the biosensor strain for QS inhibition assays. Fresh microbial colonies were suspended in sterile normal saline solution (NSS) and adjusted to an optical density corresponding to approximately 0.5 McFarland standard to obtain a uniform inoculum density prior to testing.

### 2.7 Antibacterial activity

The agar well diffusion technique was applied to assess in vitro antibacterial performance. Prepared bacterial suspensions were spread symmetrically over the surfaces of fresh agar plates using sterile cotton applicators. Wells were subsequently drilled into the agar foundation using a sterile cork borer, and each orifice was loaded with a 20 μL volume of extract. Positive and negative control benchmarks were assigned to streptomycin and 10% DMSO, respectively. Following incubation under optimal conditions, the diameters of the resulting inhibition zones were logged to document antibacterial susceptibility.

### 2.8 QS inhibitory activity

Similarly, the agar well diffusion technique was also applied to assess the QS inhibitory activity using *C. violaceum* as the biosensor strain. The standardized bacterial suspension was uniformly spread onto the surface of agar plates, and wells were prepared aseptically using a sterile cork borer. Each well was loaded with 20 μL of extract (100 mg/mL stock concentration). After incubation, plates were examined for turbid but pigmentless zones surrounding the wells, indicating inhibition of violacein production and, consequently, QS inhibition. In contrast, clear zones were interpreted as evidence of antibacterial activity. Cinnamaldehyde and 10% DMSO served as the positive and negative controls, respectively.

### 2.9 Minimum inhibitory concentration and minimum bactericidal concentration

Minimum inhibitory concentration (MIC) and minimum bactericidal concentration (MBC) limits for the extracts against Gram-positive *S. aureus* and Gram-negative *E. coli* were determined using a resazurin-based 96-well microdilution framework adapted from Padayao et al. (2025), with subtle adjustments. Suspended bacteria were brought to a 0.5 McFarland standard and diluted to working concentrations. Initially, 100 μL of Mueller–Hinton broth (MHB) was allocated into all wells of a 96-well microplate. Afterward, 100 μL of extract (50 mg/mL stock concentration) was added to the first well and serially diluted down the rows in a two-fold pattern, achieving a final concentration range spanning 50 to 0.391 mg/mL. Controls included streptomycin (1 mg/mL, positive) and DMSO (negative). Following a 24 h incubation phase, resazurin indicator dye was added to all wells to monitor metabolic viability. A distinct blue-to-pink color change signified active bacterial growth. The MIC value was documented as the lowest sample concentration capable of completely arresting this color shift. For MBC derivation, aliquots from wells showing zero color conversion were streak-inoculated onto fresh agar media to confirm the absolute destruction of viable bacterial cells.

### 2.10 Phytochemical profiling by UPLC-Q-orbitrap-ESI-MS/MS

Phytochemical profiling was performed using a Waters Cortecs C18 column (2.1 × 150 mm, 1.6 μm) set to 45 °C with a continuous solvent flow rate of 0.3 mL/min. The mobile phase system combined 0.1% FA in water (Phase A) and 0.1% FA in ACN (Phase B). The gradient steps were programmed as follows: starting at 95% A, dropping linearly to 5% A between 50 and 55 min, and re-equilibrating back to 95% A for 5 min to complete a 60 min total run. Mass spectra were acquired with a heated electrospray ionization (H-ESI) source run in both positive and negative ion capture modes, with spray voltages set at 3.5 and 3.0 kV, respectively. Gas configurations for the sheath, auxiliary, and sweep parameters were held constant at 50, 10, and 1 arbitrary units, respectively, with a capillary temperature of 320 °C. Full-scan spectra were gathered over an *m*/*z* window of 100–1500 at MS1 and MS2 resolution benchmarks of 70,000 and 17,500, respectively. Fragmentation profiles were mapped utilizing a data-dependent TopN (*n* = 10) algorithm with stepped normalized collision energy settings of 10, 30, and 50 eV. Compound identification was performed by comparing accurate mass measurements, predicted molecular formulas, retention behavior, and MS/MS fragmentation patterns with available reference standards, online spectral databases, and previously reported literature. The compounds were considered tentatively identified based on their mass accuracy and characteristic fragment ions consistent with reported fragmentation patterns of related compounds. Major diagnostic fragment ions were used to support structural annotation, particularly for flavonoids and phenolic acids. Compounds lacking sufficient spectral similarity or literature support were not assigned and were classified as unidentified peaks.

### 2.11 Phytochemical quantification by HPLC

Dried extracts and reference compounds were dissolved in HPLC-grade MeOH using brief ultrasonication and subsequently filtered through 0.45 μm PVDF syringe filters (Ma, 2024). HPLC analysis was performed using a Waters Alliance e2695 separation module equipped with a 2489 UV/Vis detector. Chromatographic separation was achieved on a YMC-Pack Pro C18 column (4.6 × 250 mm, 5 μm) maintained at 30 °C with a flow rate of 1.0 mL/min. The mobile phase consisted of 0.1% FA in water (A) and ACN (B). The gradient elution program was as follows: 95% A from 0 to 10 min, decreasing to 67% A at 20 min, then to 0% A between 40 and 45 min, followed by column re-equilibration. Detection was performed at 330 nm using an injection volume of 10 μL. Calibration curves were constructed using purified standards of kaempferol 3,7-di-*O*-rhamnoside (**1**), kaempferol 7-*O*-neohesperidoside (**2**), kaempferol 3-*O*-glucoside (**3**), kaempferol 3-*O*-rhamnoside (**4**), kaempferol 7-*O*-rhamnoside (**5**), and kaempferol (**6**) at a minimum of five concentration levels. Method validation included the determination of the limits of detection (LOD) and quantification (LOQ), while linearity was evaluated using the coefficient of determination (*R*^2^). Compound concentrations were calculated from the corresponding calibration curves and expressed as mean ± standard deviation (SD) (*n* = 3).

### 2.12 Statistical analyses

Statistical analyses were performed using GraphPad Prism 10.2.3 (GraphPad Software, Boston, MA, USA). The data were normalized prior to statistical analysis. Differences between groups were evaluated using Student's *t*-test, and statistical significance was established at *p* < 0.05. All results are presented as mean ± SD.

## 3 Results

### 3.1 TPC and TFC contents

The TPC and TFC contents of FLH and SLH extracts are shown in Fig. 3a–b. The TPC of SLH (103.0 mg TAE/g extract) was significantly higher than that of FLH (75.4 mg TAE/g extract) (**p < 0.01), indicating increased phenolic accumulation during leaf development. In contrast, the TFC of SLH (7.10 mg QE/g extract) was only slightly higher than that of FLH (6.87 mg QE/g extract), and the difference was not statistically significant. These results suggest that although total phenolic levels increased with leaf maturation, total flavonoid content remained relatively stable between the two early developmental stages.

**Fig. 3:**
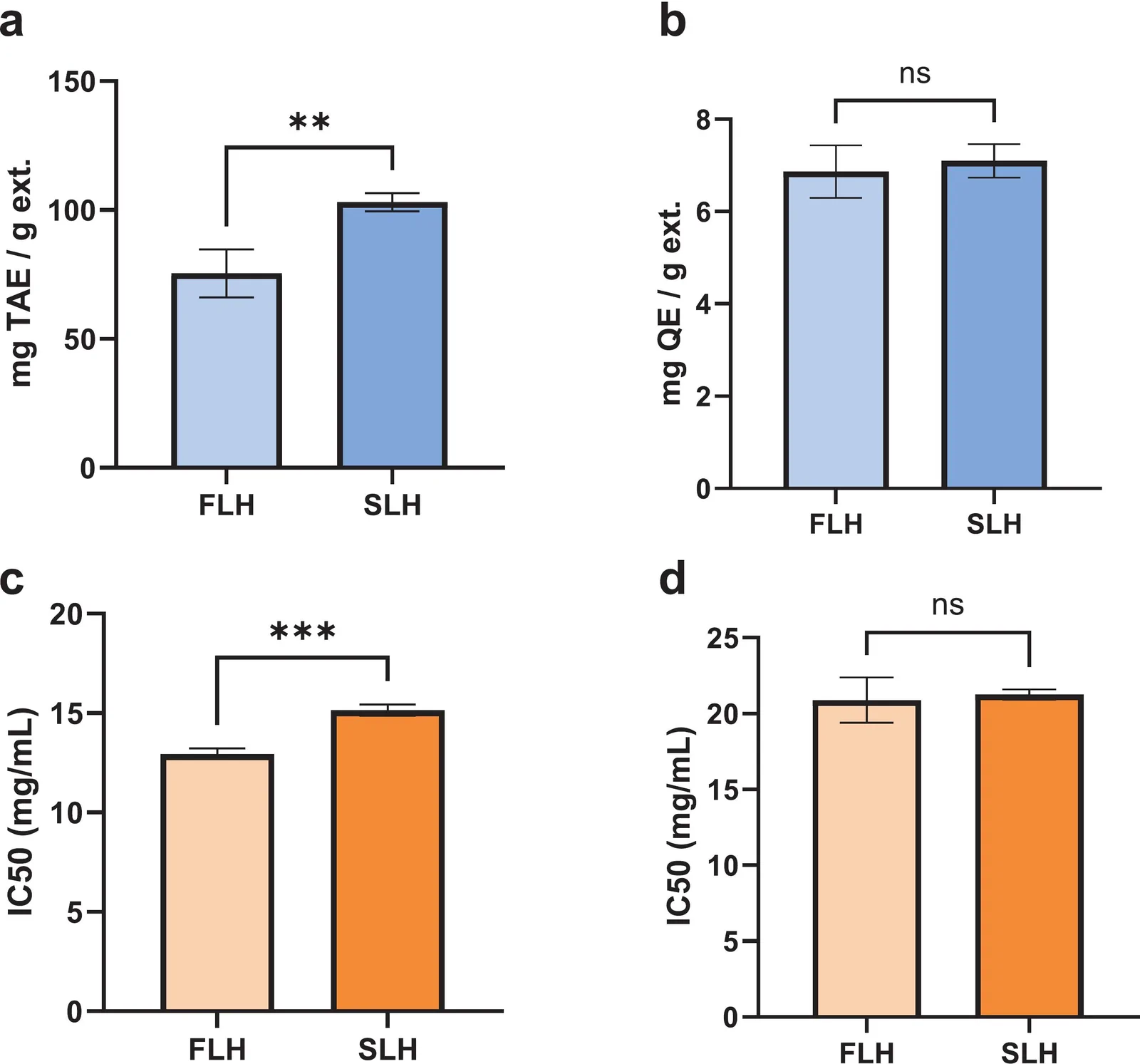
Quantitative evaluation of phytochemical and antioxidant profiles for FLH versus SLH: (a) Total Phenolic Content (TPC), (b) Total Flavonoid Content (TFC), (c) ABTS radical quenching capacity, and (d) DPPH radical scavenging performance. Data comparisons were analyzed via Student's *t*-test, with levels of significance denoted as follows: **p < 0.01; ***p < 0.001; ns, not significant.

### 3.2 Radical scavenging activity

The radical scavenging activities of FLH and SLH extracts were evaluated using ABTS and DPPH assays and expressed as IC_50_ values (Fig. 3c–d). In the ABTS assay, SLH exhibited a significantly higher IC_50_ value (15.14 mg/mL) than FLH (12.95 mg/mL) (***p < 0.001), indicating lower radical scavenging activity in the more developed leaves. In contrast, no significant difference was observed between FLH (20.89 mg/mL) and SLH (21.26 mg/mL) in the DPPH assay, suggesting comparable radical scavenging capacities between the two developmental stages.

### 3.3 Antibacterial activity

The antibacterial activities of FLH and SLH extracts were evaluated against the Gram-positive bacterium *S. aureus* and the Gram-negative bacterium *E. coli* using agar well diffusion and resazurin-based microdilution assays for MIC and MBC determination. Both extracts exhibited antibacterial activity against the tested pathogens, although the degree of activity varied depending on the bacterial species (Fig. 4a–b).

**Fig. 4:**
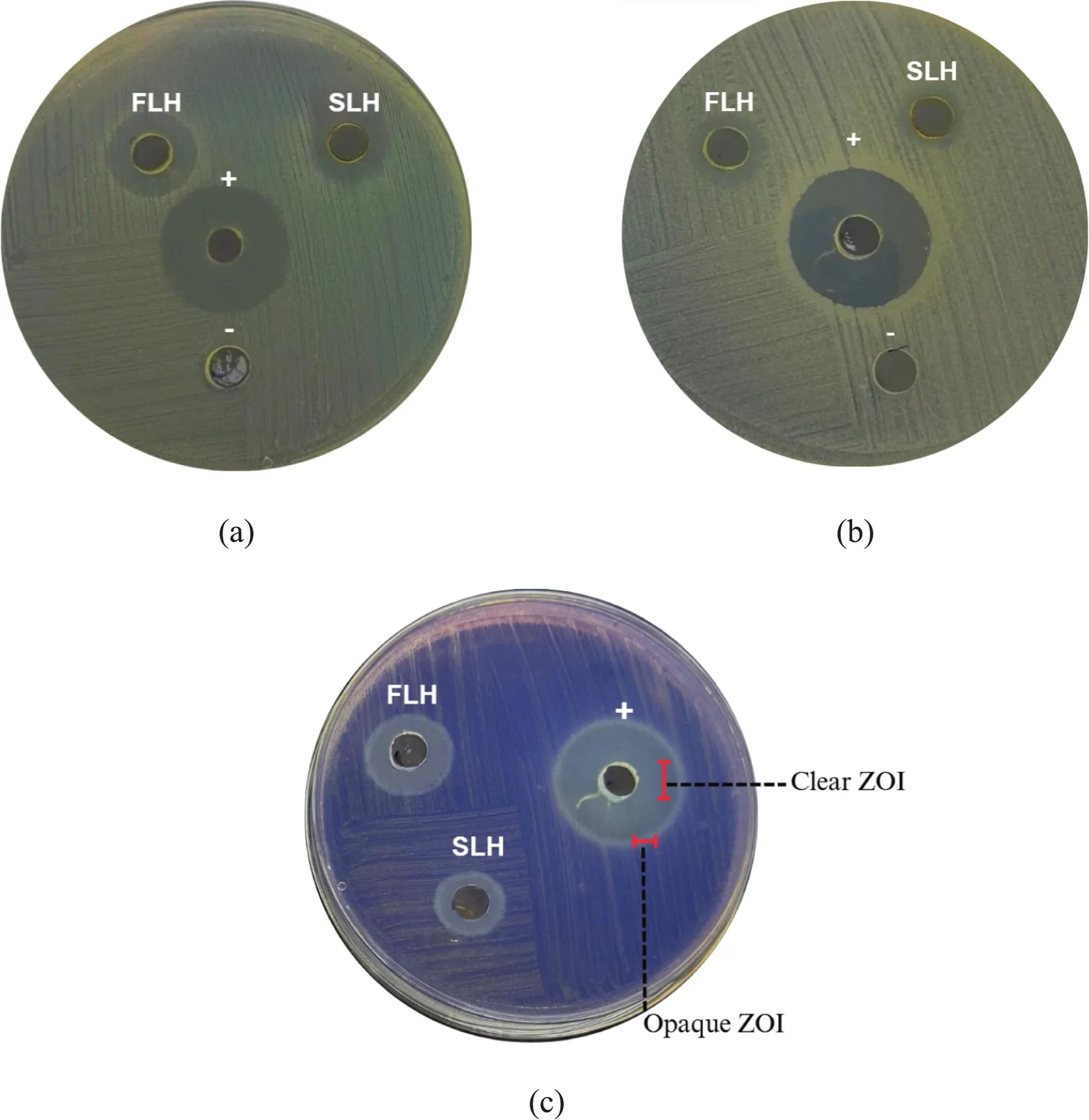
Cultured agar plates demonstrating the effects of FLH and SLH treatments inside an agar well diffusion framework. Following appropriate incubation of the seeded microbial layers, the resulting halo diameters were measured to track anti-QS properties in *C. violaceum* (c) alongside broad antibacterial trends in *E. coli* (b) and *S. aureus* (a). Benchmarks for positive and negative controls are identified by the (+) and (−) signs.

Against *S. aureus*, both extracts demonstrated strong antibacterial activity. While the positive control generated the most pronounced clear zone (22.8 mm), FLH produced an inhibition zone of 16 mm, which is statistically different compared with 14 mm for SLH (*p* < 0.05). In the microdilution assay, both extracts exhibited identical MIC and MBC values, with bacterial growth remaining inhibited at the lowest concentration tested (0.391 mg/mL); therefore, the MIC was reported as <0.391 mg/mL, while the MBC was 0.391 mg/mL. Because concentrations below 0.391 mg/mL were not evaluated, the exact MIC endpoint against *S. aureus* could not be established.

In contrast, antibacterial activity against *E. coli* was considerably lower. FLH produced an inhibition zone of 9 mm, whereas SLH produced a smaller zone of 7 mm. Consistent with the diffusion assay results, both extracts showed identical MIC and MBC values against *E. coli*, with an MIC of 12.5 mg/mL and an MBC of 25 mg/mL.

Overall, FLH consistently exhibited slightly stronger antibacterial activity than SLH in the agar well diffusion assay against both bacterial species. However, the two extracts displayed identical MIC and MBC values for each pathogen. The results further indicate that both extracts were substantially more active against *S. aureus* than against *E. coli*, as evidenced by the larger inhibition zones and lower MIC and MBC values observed for the Gram-positive bacterium.

### 3.4 QS inhibitory activity

The QS inhibitory activities of FLH and SLH extracts were evaluated against *C. violaceum* by measuring the formation of opaque anti-QS zones (Fig. 4c). Distinct zones of turbid yet unpigmented bacterial growth surrounding the wells were indicative of QS inhibition through suppression of violacein production. FLH produced a mean clear zone of 12.6 mm and a mean anti-QS zone of 2.10 mm, whereas SLH produced a mean clear zone of 10.2 mm and a mean anti-QS zone of 1.87 mm. Although FLH showed a numerically larger anti-QS zone than SLH, the difference was not statistically significant (*p* > 0.05). These observations suggest that both extracts can interfere with violacein production in *C. violaceum*. Overall, both FLH and SLH exhibited potential QS inhibitory activity, with no significant difference between the developmental stages based on the anti-QS zone assay.

### 3.5 Phytochemical profiling using UPLC-Q-orbitrap-ESI-MS/MS analysis

Based on structural parameters including exact masses, retention times, molecular formulas, precursor ions, and historical mass spectral databases, a total of 12 constituents were tentatively annotated in the FLH extract. The underlying spectroscopic mapping was performed using UPLC-Q-orbitrap-ESI-MS/MS under dual positive and negative ionization operations. The associated analytical outputs are cataloged as base peak chromatograms in Figs. 5a–b and detailed structurally in Table 2.

**Fig. 5:**
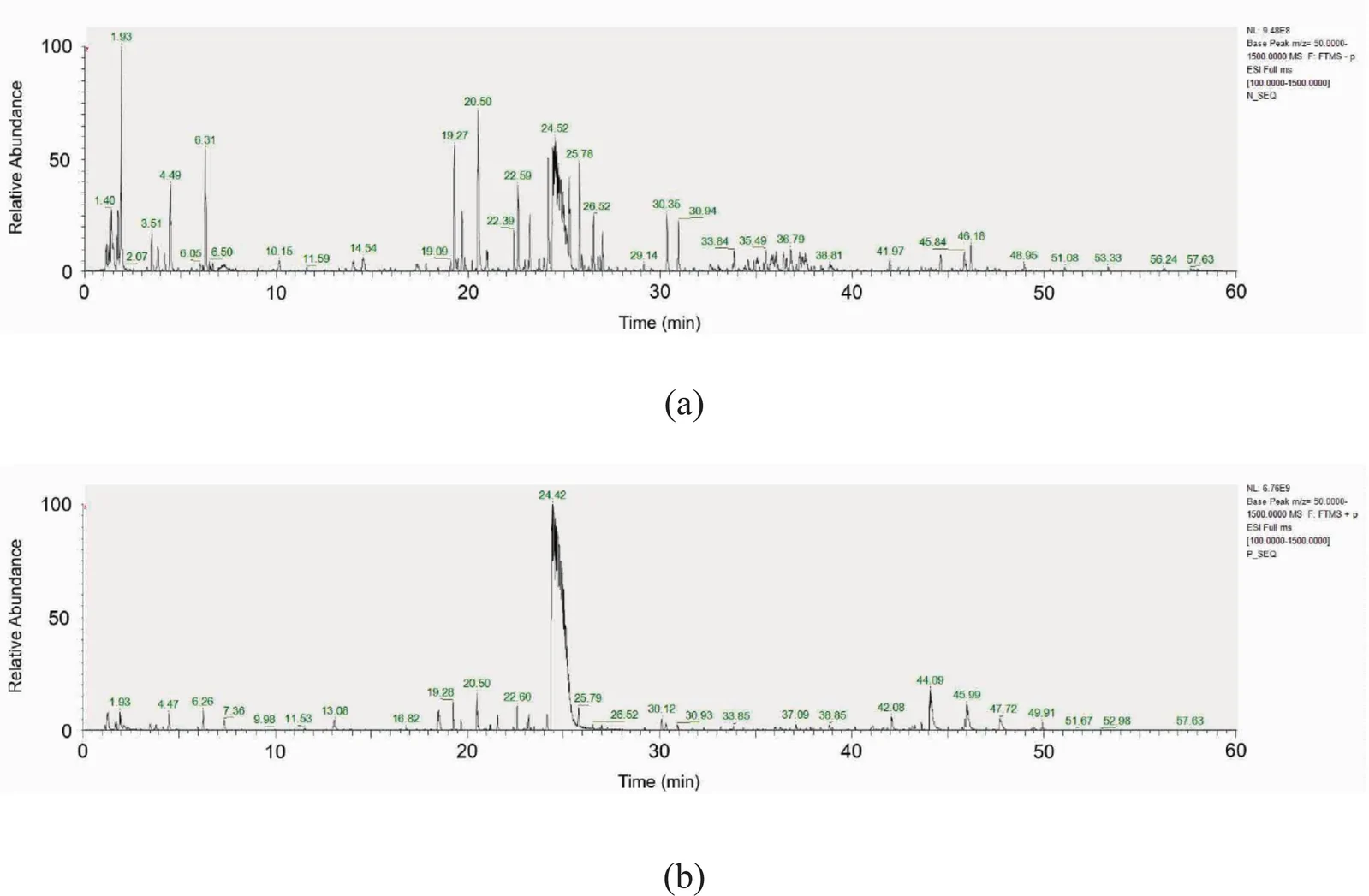
Base peak chromatograms of the FLH extract in negative (a) and positive (b) ionization modes.

The detected metabolites consisted primarily of phenolic acids and flavonoid derivatives. The identified phenolic acids included gallic acid (t_R_ = 2.57 min), caffeic acid (t_R_ = 10.15 min), *p*-coumaric acid (t_R_ = 14.54 min), and *o*-coumaric acid (t_R_ = 17.38 min), which were mainly detected in negative ionization mode as [M − H]^−^ ions. Several kaempferol glycosides were also identified, including kaempferol 7-*O*-neohesperidoside, kaempferol 3-*O*-rhamnoside 7-*O*-xyloside, kaempferol 3,7-di-*O*-rhamnoside, kaempferol 3-*O*-glucoside, kaempferol 3-*O*-rhamnoside, and kaempferol 7-*O*-rhamnoside. Additionally, datiscetin and kaempferol were detected in positive ionization mode with precursor ions at *m*/*z* 287.055 and 285.040, respectively (Fig. S1-S12).

The chromatographic profiles obtained from both ionization modes indicated that flavonoid glycosides constituted the predominant class of metabolites in the FLH extract, particularly within the retention time range of approximately 19–26 min, where several prominent peaks were observed. These findings highlight the chemical complexity of the extract and provide preliminary evidence regarding the phytochemical constituents that may contribute to its biological activities.

### 3.6 Phytochemical quantification using HPLC analysis

The target concentrations of the major flavonoids, namely kaempferol 3,7-di-*O*-rhamnoside (**1**), kaempferol 7-*O*-neohesperidoside (**2**), kaempferol 3-*O*-glucoside (**3**), kaempferol 3-*O*-rhamnoside (**4**), kaempferol 7-*O*-rhamnoside (**5**), and kaempferol (**6**), in the extracts were successfully quantified using an adapted HPLC method. Representative chromatograms of FLH and SLH extracts are shown in Fig. 6, while calibration parameters and quantified contents are summarized in Table 3. All calibration curves exhibited excellent linearity over the tested concentration range (15.63–250 μg/mL), with coefficients of determination ($R^{2}$) ranging from 0.9996 to 0.9998. The limits of detection (LOD) and quantification (LOQ) ranged from 1.013 to 7.690 μg/mL and from 3.068 to 23.302 μg/mL, respectively, demonstrating the satisfactory sensitivity of the analytical method. The obtained LOD and LOQ values varied among the analyzed compounds, reflecting differences in their detector responses; however, all quantified compounds were present at concentrations above their respective LOQs, supporting the suitability of the method for their quantitative determination.

**Fig. 6:**
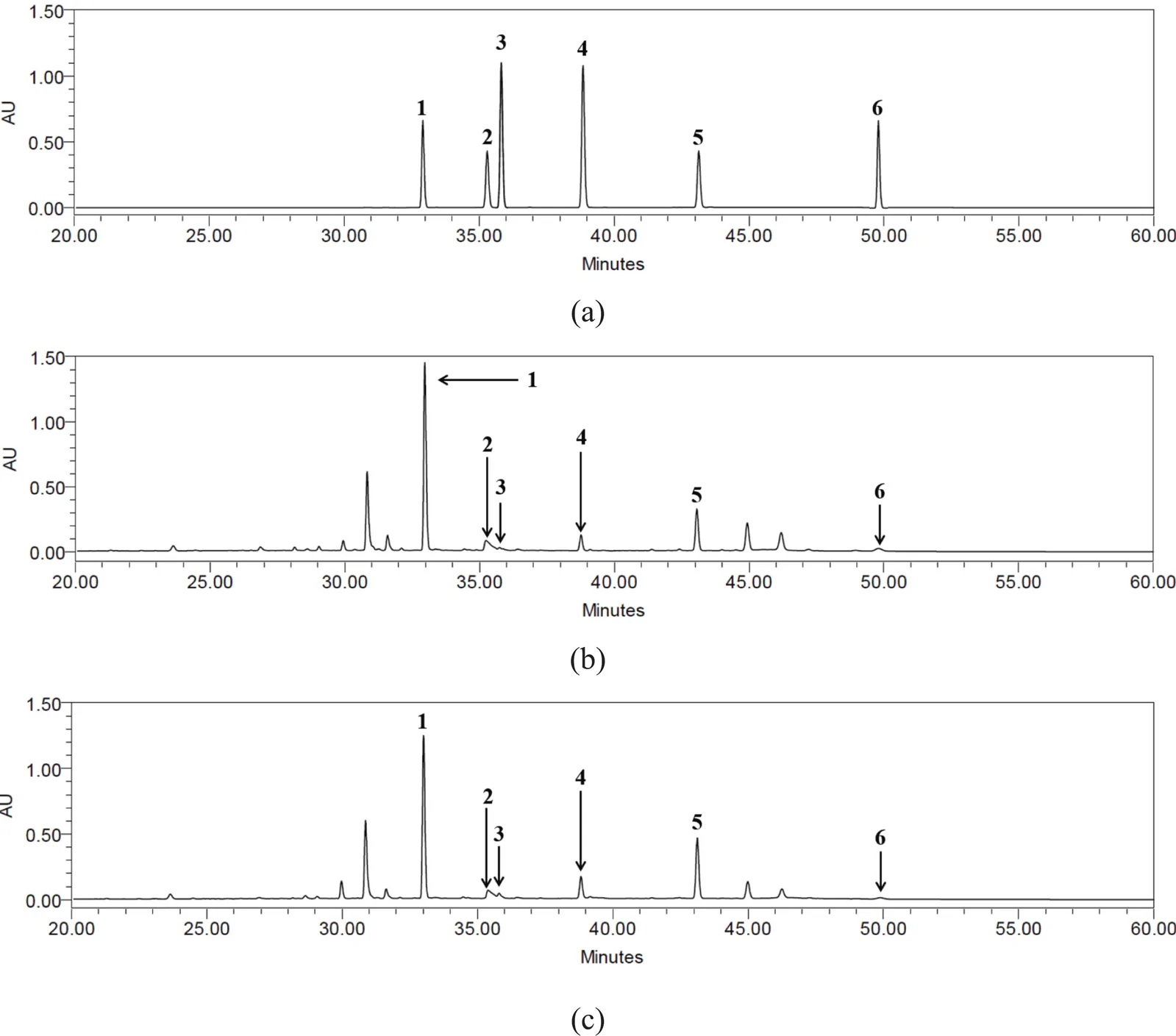
HPLC chromatograms of kaempferol 3,7-di-*O*-rhamnoside (**1**), kaempferol 7-*O*-neohesperidoside (**2**), kaempferol 3-*O*-glucoside (**3**), kaempferol 3-*O*-rhamnoside (**4**), kaempferol 7-*O*-rhamnoside (**5**), and kaempferol (**6**) (a), FLH (b), and SLH (c).

Among the quantified compounds, kaempferol 3,7-di-*O*-rhamnoside (**1**) was the predominant constituent in both extracts and was present at a higher concentration in FLH (12.89 mg/g extract) than in SLH (10.37 mg/g extract). Similarly, kaempferol 7-*O*-neohesperidoside (**2**) and kaempferol (**6**) were more abundant in FLH, with concentrations of 1.29 and 0.43 mg/g extract, respectively, compared with 1.08 and 0.08 mg/g extract in SLH. In contrast, kaempferol 3-*O*-glucoside (**3**), kaempferol 3-*O*-rhamnoside (**4**), and kaempferol 7-*O*-rhamnoside (**5**) were detected at higher levels in SLH. The concentration of kaempferol 3-*O*-glucoside (**3**) increased from 0.79 mg/g extract in FLH to 1.18 mg/g extract in SLH, while kaempferol 3-*O*-rhamnoside (**4**) increased from 0.21 to 0.54 mg/g extract. The most pronounced difference was observed for kaempferol 7-*O*-rhamnoside (**5**), which increased from 4.28 mg/g extract in FLH to 9.19 mg/g extract in SLH. Collectively, these results demonstrate substantial developmental stage-dependent differences in flavonoid composition between the two leaf stages.

## 4 Discussion

Secondary metabolite accumulation in plants is strongly influenced by developmental stage, environmental conditions, and physiological requirements (Mahajan et al., 2020). During early leaf development, dynamic metabolic changes regulate the biosynthesis and accumulation of phenolic compounds and flavonoids that contribute to antioxidant capacity and defense-related functions. In the present study, we investigated differences in the phytochemical composition and biological activities of early developmental leaf stages of *H. verticillatum*. Changes in flavonoid composition during leaf maturation may influence not only overall phytochemical accumulation but also the functional properties of plant extracts, including their antioxidant, antibacterial, and QS inhibitory activities (Chaijan et al., 2025). Characterizing these developmental shifts is crucial for identifying the precise harvesting window that maximizes the dietary health benefits and functional efficacy of this wild edible green.

The significantly higher TPC observed in SLH indicates increased accumulation of phenolic compounds during leaf maturation. Phenolic compounds are well known for their roles in protecting plants against oxidative stress and other environmental challenges, and their greater abundance in more developed leaves may reflect physiological adaptations associated with leaf development (Lin et al., 2016; Valdés-Correcher et al., 2025). In contrast, TFC did not differ significantly between FLH and SLH. Interestingly, despite its lower TPC, FLH exhibited significantly stronger ABTS radical scavenging activity than SLH, whereas no significant difference was observed in the DPPH assay. The differential responses between the two assays may be related to differences in the physicochemical properties of the radicals, including their polarity and accessibility to antioxidant constituents. Such differences can influence the relative contribution of individual phenolic and flavonoid compounds to radical-scavenging activity. Therefore, the stronger ABTS activity observed for FLH, despite its lower TPC, may reflect differences in the composition and relative abundance of specific flavonoid constituents rather than a general increase in total phenolic or flavonoid content (Jian et al., 2025). The discrepancy between the ABTS and DPPH assays may be attributed to differences in radical structure, reaction mechanisms, steric accessibility, and the solubility characteristics of individual antioxidant compounds (Wołosiak et al., 2022). In food systems, hydrophilic antioxidants often demonstrate superior performance in inhibiting lipid oxidation at interfaces. The lower IC_50_ value observed for FLH indicates greater ABTS radical-scavenging activity at the younger developmental stage compared with SLH, although the relatively high IC_50_values suggest limited activity of the crude extracts.

Both FLH and SLH exhibited antibacterial activity against *S. aureus* and *E. coli*, with stronger activity observed against the Gram-positive bacterium *S. aureus*. This difference in susceptibility is commonly attributed to structural differences in bacterial cell envelopes. Gram-negative bacteria such as *E. coli* possess an outer lipopolysaccharide membrane that limits the penetration of many phytochemicals (Lai et al., 2026), whereas Gram-positive bacteria have a comparatively more accessible peptidoglycan layer (Zhang et al., 2025). In addition to the outer-membrane barrier, the lower susceptibility of *E. coli* may also be influenced by porins and efflux pumps. Porins regulate the entry of compounds into the bacterial cell, while efflux systems such as AcrAB–TolC can remove antimicrobial compounds from the cell (Alzayn et al., 2021). These mechanisms may reduce the intracellular concentration of antibacterial constituents and contribute to the higher MIC and MBC values observed for *E. coli*. However, these mechanisms were not directly investigated in the present study and therefore warrant further mechanistic investigation.

Although FLH produced a significantly larger inhibition zone against *S. aureus* than SLH in the agar diffusion assay, both extracts exhibited identical MIC and MBC thresholds. The apparent difference between assays may reflect their distinct principles. In agar diffusion, inhibition-zone size is influenced not only by antibacterial potency but also by the diffusion, solubility, and relative abundance of diffusible constituents within the crude extracts. Thus, differences in the phytochemical composition of FLH and SLH may have influenced their diffusion through the agar and resulted in slightly different inhibition zones. In contrast, MIC and MBC were determined using two-fold serial dilutions, which provide discrete concentration endpoints. Consequently, relatively small differences in antibacterial potency between FLH and SLH may not have been sufficient to shift the MIC or MBC to the next two-fold concentration level, resulting in identical values despite the differences observed in the agar diffusion assay.

For *S. aureus*, growth remained inhibited at the lowest concentration tested (0.391 mg/mL), and the MIC was therefore reported as < 0.391 mg/mL. This value represents a threshold rather than an exact MIC because lower concentrations were not evaluated. Consequently, the precise inhibitory endpoint cannot be established from the present experiment and should not be inferred beyond the tested concentration range. However, the lower concentration threshold observed against *S. aureus* demonstrates substantially greater susceptibility of this pathogen to the extracts. The radical scavenging and antibacterial activities observed in FLH may be associated with differences in the abundance of specific flavonoid constituents. Previous studies have demonstrated that flavonoid biosynthesis and accumulation can vary substantially during leaf development, resulting in distinct phytochemical profiles at different developmental stages (Anwar et al., 2017; Jian et al., 2025). Such developmental regulation of flavonoid metabolism is consistent with the compositional differences observed between FLH and SLH in the present study, where the abundance of several kaempferol derivatives differed markedly between developmental stages.

In contrast, the relatively high concentrations required to inhibit and kill *E. coli* should be interpreted in the context of the crude nature of the extracts. Crude plant extracts contain complex mixtures of active and inactive constituents; therefore, the nominal extract concentration does not correspond to the concentration of any individual antibacterial compound. The MIC and MBC values observed against *E. coli* (12.5 and 25 mg/mL, respectively) consequently indicate limited antibacterial efficacy and should be regarded as preliminary in vitro findings rather than concentrations directly applicable to food systems (Table 1).

**Table 1:** MIC and MBC of FLH and SLH extracts against selected bacterial pathogens.

Extract	Antibacterial activity (mg/mL)			
	*S. aureus*		*E. coli*	
	MIC	MBC	MIC	MBC
FLH	< 0.391	0.391	12.5	25
SLH	< 0.391	0.391	12.5	25

**Table 2:** Tentatively identified compounds in FLH extract by UPLC-Q-Orbitrap-ESI-MS/MS analysis in negative and positive ionization modes.

t_R_ (min) a	Adducts	Precursor *m/z*	Δ*m*/*z* ppm ^***b***^	Exact mass (Da)	Molecular formula	Tentative identity
2.57	[M-H]^−^	169.013	4.96	170.022	C_7_H_6_O_5_	Gallic acid
10.15	[M-H]^−^	179.035	4.53	180.042	C_9_H_8_O_4_	Caffeic acid
14.54	[M-H]^−^	163.040	6.16	164.047	C_9_H_8_O_3_	*p*-Coumaric acid
17.38	[M-H]^−^	163.040	6.75	164.047	C_9_H_8_O_3_	*o*-Coumaric acid
19.27	[M-H]^−^/[M + H]^+^	593.151/595.166	0.10/1.33	594.158	C_27_H_30_O_15_	Kaempferol 7-*O*-neohesperidoside
19.67	[M-H]^−^/[M + H]^+^	563.141/565.155	0.02/1.36	564.148	C_26_H_28_O_14_	Kaempferol 3-*O*-rhamnoside 7-*O*-xyloside
20.51	[M-H]^−^/[M + H]^+^	577.176/579.170	1.17/1.82	578.158	C_27_H_30_O_14_	Kaempferol 3,7-di-*O*-rhamnoside
21.00	[M-H]^−^	447.093	0.03	448.101	C_21_H_20_O_11_	Kaempferol 3-*O*-glucoside
22.39	[M-H]^−^	431.098	0.62	432.106	C_21_H_20_O_10_	Kaempferol 3-*O*-rhamnoside
24.17	[M-H]^−^/[M + H]^+^	431.098	0.69/1.68	432.106	C_21_H_20_O_10_	Kaempferol 7-*O*-rhamnoside
25.27	[M + H]^+^	287.055	0.98	286.048	C_15_H_10_O_6_	Datiscetin
25.91	[M-H]^−^/[M + H]^+^	285.040/287.055	0.49/1.34	286.048	C_15_H_10_O_6_	Kaempferol

a Retention time. Only the predefined confidence threshold of ID score ≥ 0.7 was retained. ^*b*^ Δ*m*/*z* ppm was calculated from unrounded observed *m*/*z* values using full software precision, whereas the *m*/*z* values reported in the table were rounded to three decimal places; Δppm was calculated as follows: Δppm = (observed *m*/*z* − calculated *m*/*z*)/calculated *m*/*z* × 10^6^.

**Table 3:** Calibration data and contents of compounds **1**–**6** in FLH and SLH extracts.

Compound	Calibration data						Content (mg/g extract)	
	t_R_ (min) ^*a*^	Range (μg/mL)	Calibration equation	*R*^2 *b*^	LOD ^*c*^ (μg/mL)	LOQ ^*d*^ (μg/mL)	FLH	SLH
**1**	32.85	15.63–250	y = 26,033× + 145,221	0.9998	4.095	12.412	12.89 ± 0.11	10.37 ± 0.15
**2**	35.47	15.63–250	y = 33,097× - 33,509	0.9996	3.308	10.025	1.29 ± 0.03	1.08 ± 0.05
**3**	35.82	15.63–250	y = 12,799× + 8457.7	0.9997	1.463	4.432	0.79 ± 0.01	1.18 ± 0.01
**4**	38.74	15.63–250	y = 28,840× + 156,255	0.9998	2.658	7.782	0.21 ± 0.01	0.54 ± 0.15
**5**	43.47	15.63–250	y = 12,506× + 74,631	0.9997	1.013	3.068	4.28 ± 0.17	9.19 ± 0.22
**6**	49.81	15.63–250	y = 30,451× + 7964	0.9998	7.690	23.302	0.43 ± 0.02	0.08 ± 0.02
Total							19.89	22.44

^*a*^ Retention time; ^*b*^ Coefficient of determination; ^*c*^ Limit of detection; ^*d*^ Limit of quantification. Compounds: kaempferol 3,7-di-*O*-rhamnoside (**1**), kaempferol 7-*O*-neohesperidoside (**2**), kaempferol 3-*O*-glucoside (**3**), kaempferol 3-*O*-rhamnoside (**4**), kaempferol 7-*O*-rhamnoside (**5**), and kaempferol (**6**).

In addition to their antibacterial effects, both extracts exhibited QS inhibitory activity against *C. violaceum*, as evidenced by the formation of pigmentless opaque zones indicative of violacein inhibition (Kadam et al., 2021). FLH produced a slightly larger anti-QS zone than SLH, suggesting developmental differences in the phenotypic response observed in the assay. In food science, QS is a critical mechanism regulating both the expression of virulence factors in foodborne pathogens and the phenotypes responsible for bacterial food spoilage and biofilm formation on food-processing surfaces (Alum et al., 2025). Plant-derived QS inhibitors have been explored as complementary strategies for food preservation because disruption of QS may help limit spoilage-associated phenotypes, biofilm formation, and foodborne pathogenicity, with potential benefits for food safety and shelf-life extension (Bai & Vittal, 2014; Machado et al., 2019). These findings consequently position *H. verticillatum* extracts as a viable source of plant-derived agents for innovative anti-biofilm and antimicrobial approaches based on their documented anti-QS performance. Although isolating individual bioactive components and verifying their practical efficacy requires extensive future validation, this study establishes baseline evidence of the extract's functional value as a health-promoting dietary crop.

UPLC-Q-orbitrap-ESI-MS/MS profiling revealed that the extracts were predominantly composed of kaempferol glycosides, including kaempferol 3,7-di-*O*-rhamnoside (**1**), kaempferol 3-*O*-glucoside (**3**), kaempferol 3-*O*-rhamnoside (**4**), and kaempferol 7-*O*-rhamnoside (**5**). These compounds belong to flavonoid classes that have been widely reported to possess antioxidant, antimicrobial, and QS inhibitory activities (Ullah et al., 2020; Nascimento et al., 2025). The dominance of kaempferol glycosides in *H. verticillatum* suggests that its flavonoid biosynthesis heavily favors the kaempferol branch of the phenylpropanoid pathway, specifically at the critical dihydrokaempferol branch point (Gifford et al., 2018). While dihydroflavonol intermediates can be hydroxylated toward the quercetin branch of the flavonoid biosynthetic pathway by flavonoid 3′-hydroxylase (F3’H), the predominance of kaempferol derivatives in *H. verticillatum* is consistent with a greater contribution of the kaempferol branch to the observed flavonoid profile (Mao et al., 2025). The simultaneous occurrence of mono- and diglycosylated flavonoids is also consistent with the activity of uridine diphosphate-dependent glycosyltransferases (UGTs), which can catalyze glycosylation at different hydroxyl positions of the flavonoid backbone (Singh et al., 2017). However, the involvement or differential regulation of F3’H, UGTs, or other biosynthetic enzymes was not directly examined in the present study and therefore cannot be confirmed from the metabolite data alone. From an eco-physiological perspective, the predominance of kaempferol derivatives may be associated with photoprotective and stress-response functions reported for kaempferol-related compounds in plants (Liu et al., 2021; Shojaie et al., 2016). These compounds are highly prized dietary antioxidants linked to reduced systemic inflammation and chronic disease risk, validating the traditional consumption of this genus as a functional vegetable. Similar glycosylation patterns have been reported in related *Hylotelephium* species. In *H. sieboldii*, Iwashina et al. (2022) reported several kaempferol and quercetin glycosides containing glucose and/or rhamnose residues, including 3-*O*-glucosides, 3-*O*-rhamnosides, 3-*O*-glucoside-7-*O*-rhamnosides, and 3,7-di-*O*-rhamnosides. In *H. telephium*, Arvia et al. (2024) similarly reported that kaempferol and quercetin glycosides, particularly di- and tri-glycosylated derivatives, were predominant, although definitive identification of individual glycosides was limited by the lack of reference standards. These findings support the occurrence of diverse glycosylated flavonoids within the genus, although the specific glycosylation patterns may vary among species. However, the molecular mechanisms underlying the accumulation of these compounds in *H. verticillatum* remain unclear. Further studies involving transcriptomic analyses and enzyme characterization will be necessary to elucidate the regulatory processes governing this metabolic profile.

HPLC quantification further revealed distinct differences in flavonoid glycosylation profiles between the developmental stages of *H. verticillatum*. The diglycosides kaempferol 3,7-di-*O*-rhamnoside (**1**) and kaempferol 7-*O*-neohesperidoside (**2**) were more abundant in FLH, whereas monoglycosylated derivatives such as kaempferol 3-*O*-glucoside (**3**), kaempferol 3-*O*-rhamnoside (**4**), and kaempferol 7-*O*-rhamnoside (**5**) accumulated at higher levels in SLH. Previous studies have suggested that certain flavonoids can interfere with QS signaling pathways through interactions with regulatory proteins such as CviR (Gerdt & Blackwell, 2014). However, the present study assessed QS inhibition phenotypically through violacein suppression and did not investigate specific QS receptors or signaling pathways. Therefore, any relationship between individual flavonoids and the observed QS inhibitory activity should be considered preliminary and requires confirmation using isolated compounds and targeted mechanistic assays. In contrast, kaempferol (**6**) was detected at higher levels in FLH. These developmental differences suggest dynamic regulation of flavonoid glycosylation during early leaf ontogeny. Because glycosylation influences flavonoid stability, transport, solubility, and biological activity, variations in glycosylation profiles may contribute to the distinct biological properties observed between FLH and SLH extracts (Krawczyk-Łebek et al., 2025). Young leaves are generally more susceptible to environmental stress, herbivory, and microbial attack because their structural defense systems are not yet fully developed. Consequently, early developmental stages often rely more heavily on chemical defense mechanisms, including the accumulation of flavonoid glycosides and other protective secondary metabolites (Anjali et al., 2023). Crucially, this early stage corresponds to when the leaves are exceptionally soft, tender, and most palatable for fresh salad consumption. The higher abundance of diglycosylated flavonoids in early-stage harvests (FLH) may also have practical implications for the utilization of *H. verticillatum* as a source of bioactive compounds. Flavonoid glycosylation is generally associated with enhanced water solubility and can influence chemical stability, characteristics that may affect their behavior and bioavailability in biological systems. However, the present study did not directly evaluate bioavailability or product-formulation performance. Therefore, the predominance of diglycosylated kaempferol derivatives in FLH may be of interest for further investigation of their potential applications in food, pharmaceutical, and cosmetic formulations. Kaempferol glycosides, particularly kaempferol 3,7-di-*O*-rhamnoside (**1**), have been associated with a range of biological activities (Ramos-Hernández et al., 2017), although the contribution of these individual compounds to the activities observed in the present extracts cannot be established from the current data. Nevertheless, further studies are needed to evaluate the individual bioactivities, mechanisms of action, physicochemical properties and application potential of these compounds in specific product systems.

Although the biosynthesis of flavonoid monoglycosides has been extensively studied, the formation and regulation of flavonoid diglycosides and other highly glycosylated derivatives remain comparatively less understood (Xie et al., 2022). Flavonol glycosides are recognized as important defense-related metabolites and often accumulate in response to environmental factors, particularly changes in light intensity and UV-B radiation (Qin et al., 2024). For example, Xie et al. (2022) reported that UV-B treatment induced the accumulation of diglycosylated flavonols in peach, suggesting that glycosylation may play an important role in flavonoid-mediated photoprotection. However, flavonoid responses to UV-B exposure are highly species-specific, and different plant species may rely on distinct flavonoid derivatives as their primary photoprotective metabolites (Neugart et al., 2021). Therefore, although the higher abundance of diglycosides observed in FLH may reflect developmental regulation of flavonoid glycosylation, the precise physiological roles of these compounds in *H. verticillatum* remain unclear.

Furthermore, several unidentified peaks exhibited UV spectral characteristics similar to those of kaempferol glycosides, suggesting the presence of additional structurally related flavonoid derivatives (Luan et al., 2016). However, these peaks could not be confidently assigned because sufficient MS/MS evidence or authentic reference standards were unavailable. Among these, one prominent unidentified peak was tentatively assigned as kaempferol 3-*O*-rhamnoside 7-*O*-xyloside based on its chromatographic behavior and mass spectral features, although definitive identification was not possible because an authentic reference standard was unavailable. The observed developmental differences in the abundance of these glycosides further support the notion that leaf maturation influences flavonoid glycosylation patterns in *H. verticillatum*. Such changes may contribute to the distinct phytochemical profiles and biological activities observed between FLH and SLH. Further studies involving compound isolation, glycosyltransferase characterization, transcriptomic analysis, and controlled environmental experiments are needed to elucidate the regulatory mechanisms underlying these developmental changes.

The correlation and hierarchical clustering analyses further supported the close relationship between developmental stage, flavonoid composition, and biological activity in *H. verticillatum*. The clear separation of FLH and SLH samples based on both metabolite composition and functional activities suggests substantial metabolic reprogramming during early leaf development. Notably, antibacterial and QS inhibitory activities clustered more closely with specific kaempferol derivatives than with TPC. These findings further emphasize the importance of developmental regulation of flavonoid biosynthesis and glycosylation in determining the biological properties of *H. verticillatum* extracts.

The findings of this study indicate that early leaf ontogeny in *H. verticillatum* is accompanied by substantial qualitative changes in flavonoid composition that significantly influence biological activity. Although SLH accumulated higher TPC, FLH consistently exhibited stronger radical scavenging, antibacterial, and QS inhibitory activities, suggesting that these effects are more closely associated with the relative abundance of specific flavonoid constituents than with total phenolic accumulation alone. Given that the seedlings were harvested approximately 20 days after germination, the enhanced biological activities observed in FLH may reflect the greater reliance of young leaves on chemical defense mechanisms during early development. From an applied perspective, these results suggest that early-stage leaves may represent a promising source of bioactive flavonoids with potential value for functional food, pharmaceutical, and cosmetic applications. From a food-systems perspective, these results confirm that harvesting *H. verticillatum* at its ultra-early vegetative stage yields a tender salad green with peak functional and health-promoting properties. Furthermore, the observed developmental variation in phytochemical composition highlights the importance of harvest timing as an agricultural determinant of the phytochemical quality of *H. verticillatum*. Based on the present findings, harvesting at approximately 20 days post-germination represents a promising early harvest stage for obtaining the phytochemical and bioactive characteristics observed in this study, although further evaluation across additional developmental stages is required to establish an optimal harvest window.

## 5 Conclusions

The present study provides new insights into the developmental regulation of flavonoid biosynthesis in *H. verticillatum* and highlights the importance of qualitative phytochemical variation in determining functional bioactivity during early plant development. The pronounced bioactivity and rich kaempferol glycoside profile of early-stage leaves indicate that *H. verticillatum* has considerable potential as a highly nutritious, wild edible salad green and a valuable source of functional food ingredients, natural antioxidants, and dietary biopreservatives. Although several metabolites were tentatively identified based on UPLC-Q-orbitrap-ESI-MS/MS data, further structural confirmation through compound isolation and NMR analysis is required to fully characterize the unidentified constituents. Future studies should also evaluate the biological activities of individual compounds and assess their stability under typical food processing and digestion conditions to identify those primarily responsible for the observed bioactivities. In addition, investigations into flavonoid biosynthetic regulation and the activities of isolated metabolites would provide deeper insight into the metabolic and functional significance of kaempferol derivatives in *H. verticillatum* as a dietary source.

## CRediT authorship contribution statement

**Neil Patrick Uy:** Writing – original draft, Investigation, Formal analysis. **Reyna Marie Therese Sanchez:** Validation, Investigation. **Sang-Yun Lee:** Investigation, Data curation. **Jonie Yee:** Supervision, Data curation. **Chung-Ho Choi:** Writing – original draft, Resources, Investigation, Data curation. **Sanghyun Lee:** Writing – review & editing, Supervision, Project administration, Funding acquisition, Conceptualization.

## Declaration of competing interest

The authors declare that they have no known competing financial interests or personal relationships that could have appeared to influence the work reported in this paper.

## Data Availability

All data supporting the findings of this study are available within the paper and its Supplementary Materials.
