# Supplementary material for: Developmental stage-dependent changes in kaempferol derivatives and associated antioxidant, antibacterial, and quorum sensing inhibitory activities in Hylotelephium verticillatum leaves
Source: Food Chem X. 2026 Sep 25;39:104520. doi: 10.1016/j.fochx.2026.104520 (PMC13636310; doi:10.1016/j.fochx.2026.104520)
Supplement: Supplementary file 1 [file mmc1.docx]

**Supplementary Materials**

**Fig S1.** MS2 spectrum of gallic acid.

**Fig S2.** MS2 spectrum of caffeic acid.

**Fig S3.** MS2 spectrum of *p*-coumaric acid.

**Fig S4.** MS2 spectrum of *o*-coumaric acid.

**Fig S5.** MS2 spectrum of kaempferol 7-*O*-neohesperidoside.

**Fig S6.** MS2 spectrum of kaempferol 3-*O*-rhamnoside 7-*O*-xyloside.

**Fig S7.** MS2 spectrum of kaempferol 3,7-di*-O-*rhamnoside.

**Fig S8.** MS2 spectrum of kaempferol 3-*O-*glucoside.

**Fig S9.** MS2 spectrum of kaempferol 3-*O-*rhamnoside.

**Fig S10.** MS2 spectrum of kaempferol 7-*O-*rhamnoside.

**Fig S11.** MS2 spectrum of datiscetin.

**Fig S12.** MS2 spectrum of kaempferol.
